# Probenecid Pre-treatment Downregulates the Kidney Cl^-^/HCO_3_^-^ Exchanger (Pendrin) and Potentiates Hydrochlorothiazide-Induced Diuresis

**DOI:** 10.3389/fphys.2018.00849

**Published:** 2018-07-11

**Authors:** Sharon Barone, Jie Xu, Kamyar Zahedi, Marybeth Brooks, Manoocher Soleimani

**Affiliations:** ^1^Department of Medicine, University of Cincinnati, Cincinnati, OH, United States; ^2^Research Services, VA Medical Center, Cincinnati, OH, United States; ^3^Center on Genetics of Transport and Epithelial Biology, University of Cincinnati, Cincinnati, OH, United States

**Keywords:** Na^+^-Cl^-^ co-transporter, pendrin, collecting duct, distal convoluted tubule, salt

## Abstract

**Background:** Probenecid is a uricosuric agent that in addition to exerting a positive ionotropic effect in the heart, blocks the ATP transporter Pannexin 1 and inhibits the Cl^-^/HCO_3_^-^ exchanger, pendrin. In the kidney, pendrin blunts the loss of salt wasting secondary to the inhibition of the thiazide-sensitive Na^+^-Cl^-^ co-transporter (NCC/SLC12A3).

**Hypothesis:** Pre-treatment with probenecid down-regulates pendrin; therefore, leaving NCC as the main salt absorbing transporter in the distal nephron, and hence enhances the hydrochlorothiazide (HCTZ)-induced diuresis.

**Methods:** Daily balance studies, blood and urine chemical analysis, immunofluorescence, as well as western and northern blot analyses were utilized to examine the effects of probenecid alone (at 250 mg/kg/day) or in combination with HCTZ (at 40 mg/kg/day) on kidney function and on salt and water transporters in the collecting duct.

**Results:** Male Sprague Dawley rats were subjected to three different protocols: (1) HCTZ for 4 days, (2) probenecid for 10 days, and (3) primed with probenecid for 6 days followed by probenecid and HCTZ for 4 additional days. Treatment protocol 1 (HCTZ for 4 days) only mildly increased the urine volume (U Vol) from a baseline of 9.8–13.4 ml/day. In response to treatment protocol 2 (probenecid for 10 days), U Vol increased to 15.9 ml/24 h. Treatment protocol 3 (probenecid for 6 days followed by probenecid and HCTZ for 4 additional days) increased the U Vol to 42.9 ml/day on day 4 of co-treatment with HCTZ and probenecid (compared to probenecid *p* = 0.003, *n* = 5 or HCTZ alone *p* = 0.001, *n* = 5). Probenecid treatment at 250 mg/kg/day downregulated the expression of pendrin and led to a decrease in AQP2 expression. Enhanced diuresis by probenecid plus HCTZ was not associated with volume depletion.

**Conclusion:** Probenecid pre-treatment downregulates pendrin and robustly enhances diuresis by HCTZ-mediated NCC inhibition in kidney.

## Introduction

The distal nephron, including the distal convoluted tubule (DCT), connecting tubule (CNT) and the collecting duct (CD) plays an important role in adjusting the magnitude of salt excretion via the action of specific salt transporters, including the Na–Cl cotransporter (NCC), the epithelial sodium channel (ENaC), and the Cl^-^/HCO_3_^-^ exchanger pendrin, with NCC functioning independently and ENaC and pendrin working in tandem (Gesek and Friedman, 1995; Delpire et al., 1996; Schultheis et al., 1998; Câmpean et al., 2001; Royaux et al., 2001; Soleimani et al., 2001; Wang et al., 2001; Kim et al., 2002; Ellison, 2003; Petrovic et al., 2003; Verlander et al., 2003; Wall et al., 2003, 2004; Loffing et al., 2004; Gamba, 2005; Vallet et al., 2006; Amlal et al., 2010; Reilly et al., 2010; Sica et al., 2011). The inactivation or inhibition of NCC or pendrin does not cause any overt salt wasting under baseline conditions (Schultheis et al., 1998; Verlander et al., 2003; Wall et al., 2004; Alshahrani and Soleimani, 2017; Alshahrani et al., 2017). However, their simultaneous ablation or inactivation causes severe salt wasting in rodents, indicating an important role for pendrin in compensatory salt absorption in the setting of NCC inhibition (Soleimani et al., 2012).

In addition to salt reabsorption, the cortical collecting duct (CCD) plays a crucial role in systemic acid-base homeostasis by secreting acid or bicarbonate into the lumen via specialized intercalated cells, with A-intercalated cells being responsible for acid secretion via apical H^+^-ATPase and B-intercalated cells mediating bicarbonate extrusion into the lumen in exchange with luminal chloride via pendrin (Royaux et al., 2001; Soleimani et al., 2001; Wang et al., 2001; Verlander et al., 2003; Amlal et al., 2010; Soleimani, 2015). Na^+^ reabsorption in CCD and the CNT is *trans*-cellular and mediated primarily by ENaC, which is located on the apical membrane of principal cells (Gesek and Friedman, 1995; Loffing et al., 2004; Soundararajan et al., 2010; Svenningsen et al., 2015).

Probenecid is a uricosuric agent that inhibits the organic anion transporters (OATs) in the proximal tubule and is used in the treatment of hyperuricemia and gout (Bach and Simkin, 2014; Richette et al., 2014). In addition, probenecid has several effects of undetermined impact on kidney physiology, including the inhibition of Pannexin 1, which is an ATP transporter in the proximal tubule and the collecting duct (Silverman et al., 2008), as well as inhibition of alpha adrenergic receptors (Halpin and Renfro, 1996). Probenecid was initially developed for the prevention of renal excretion of antibiotics (Halpin and Renfro, 1996; Silverman et al., 2008; Bach and Simkin, 2014; Richette et al., 2014). It has also been shown to inhibit pendrin in mammary gland cells (Rillema and Hill, 2003). Detailed studies on cardiac function indicated that probenecid exhibits a positive ionotropic effect in the heart, specifically after reperfusion injury, and can increase the cardiac output (Koch et al., 2012; Rubinstein et al., 2014).

**FIGURE 1:**
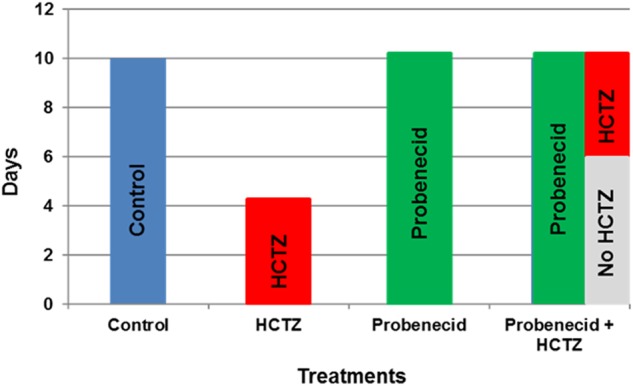
The experimental protocols examining the effects of probenecid and HCTZ on kidney function. The explanatory time course of probenecid, HCTZ and probenecid plus HCTZ treatment on kidney function in rats.

The diuretic effect of HCTZ, a specific inhibitor of NCC, is significantly blunted consequent to compensatory salt absorption by pendrin (Zahedi et al., 2013; Patel-Chamberlin et al., 2016). Based on the inhibitory effect of probenecid on pendrin (Rillema and Hill, 2003), we tested the hypothesis that pre-treatment with probenecid will downregulate or inactivate pendrin; therefore, leaving NCC as the main salt absorbing transporter in the distal nephron, thus significantly enhancing the diuretic effect of HCTZ. Toward this goal, rats were treated with HCTZ, probenecid or a combination of probenecid and HCTZ (experimental protocols described in **Figure 1**). Balance studies were performed to ascertain the effect of probenecid alone or probenecid plus HCTZ combination on salt excretion.

## Results

The time lines for experimental protocols are described in **Figure 1**. The dosing for probenecid was selected based on published literature in rodents (Emanuelsson and Paalzow, 1988; Hesselink et al., 1999). Male Sprague Dawley rats were treated under 3 different protocols: (1) HCTZ (40 mg/kg/day for 4 days), (2) probenecid (250 mg/kg/day for 10 days), and (3) primed with probenecid (250 mg/kg/day) for 6 days followed by probenecid (250 mg/kg/day) and HCTZ (40 mg/kg/day) for 4 additional days.

### Effect of Probenecid on HCTZ-Induced Diuresis

**FIGURE 2:**
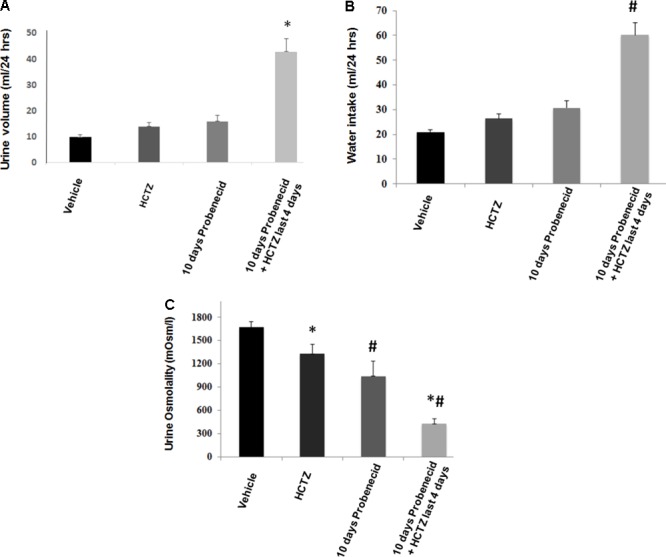
Effect of probenecid at 250 mg/kg, with or without HCTZ, on urine volume and water intake. **(A)** Effect of probenecid and probenecid/HCTZ on urine volume. Rats (5/group) were placed in metabolic cages and after acclimation treated with vehicle (control), HCTZ (4 days), probenecid at 250 mg/kg/24 h (10 days) or probenecid (250 mg/kg/24 h) for 6 days followed by probenecid and HCTZ for 4 additional days. Urine was collected daily and the urine volume was expressed as ml/24 h. **(B)** Effect of probenecid and probenecid/HCTZ on Water Intake. Measurement of water intake in rats treated with probenecid for 10 days vs. control. For comparison, water intake in rats pre-treated with probenecid and then treated with probenecid and HCTZ is shown. **(C)** Effect of HCTZ and probenecid on urine osmolality. ^∗, #^ denotes significance between Vehicle vs Probenecid/HCTZ treatment. Urine osmolality measurement in rats treated with HCTZ (40 mg/kg), 10 days of probenecid (250 mg/kg) and a co-treatment with probenecid/HCTZ.

As shown in **Figure 2A**, daily treatment with probenecid (250 mg/kg) for 10 days causes mild diuresis in rats, with urine output increasing from 9.83 ± 0.93 ml/24 h at baseline to 15.89 ± 2.21 ml/24 h (*p* = 0.01, *n* = 5). Daily administration of HCTZ (40 mg/kg/24 h for 4 days) appears to cause a very mild diuresis, with urine output increasing to 13.80 ± 2.1 ml/24 h on day 4 (*p* = 0.13, vs. baseline, *n* = 5). However, in rats that were primed with probenecid for 6 days, further treatment with HCTZ and probenecid for 4 additional days increased the urine output to 42.90 ± 4.94 ml/24 h on day 4 of probenecid/HCTZ co-administration (*p* = 0.003, *n* = 5). The results of urine volumes when corrected for body weight (ml/24 h/g body weight) are as follows: 0.045 +/- 0.004 (vehicle), 0.06 +/- 0.007 (HCTZ), 0.08 +/- 0.002 (probenecid), and 0.19 +/- 0.03 (probenecid/HCTZ), respectively, with probenecid/HCTZ treated rats showing significant differences vs. the 3 other groups (*p* < 0.004 vs. all three other groups, *n* = 5–7 in each group).

Water intake increased in rats primed with probenecid and then treated with probenecid/HCTZ combination, corresponding with enhanced urine output in this group (**Figure 2B**). **Figure 2C** depicts urine osmolality in various experimental groups and demonstrates a significant (*p* = 0.000001) reduction in urine osmolality of probenecid/HCTZ-treated rats (429 ± 61.83 mOsm/l) compared to vehicle-treated animals (1673 ± 72.84 mOsm/l).

### Effect of Probenecid, With or Without HCTZ on Kidney Function

**FIGURE 3:**
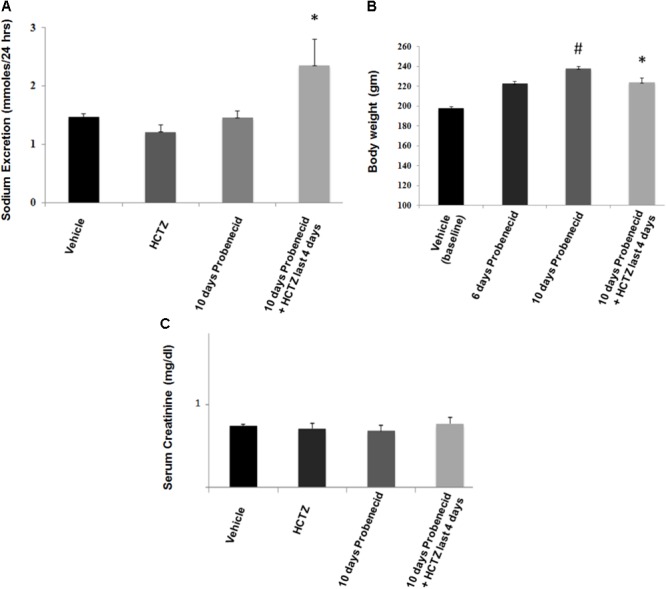
Effect of probenecid (250 mg/kg/day) with or without HCTZ, on kidney function. **(A)** Effect of probenecid with or without HCTZ, on salt excretion. Sodium excretion (mmoles/24 h) increased in probenecid primed rats that were treated with probenecid/HCTZ but not in rats treated with HCTZ or probenecid alone. ^∗^ denotes significance vs. all other groups. **(B)** Body weight measurements in probenecid and Probenecid/HCTZ treated rats. Probenecid-primed animals that were co-treated with probenecid and HCTZ for 4 additional days showed a lower body weight compared to animals that were treated only with probenecid. ^#^ denotes significance between Vehicle vs. 10 days Probenecid. **(C)** Serum creatinine levels in probenecid and probenecid/HCTZ treated animals. Serum creatinine levels (mg/dl) are not significantly different in probenecid (0.68 ± 0.04, *n* = 5), HCTZ (0.71 ± 0.10) and probenecid/HCTZ-treated groups (0.77 ± 0.05, *n* = 5) and Vehicle-treated rats (0.74 ± 0.04, *n* = 5) (*p* > 0.05 between groups).

Data depicted in **Figure 3A** indicate that the magnitude of sodium excretion increased significantly in the probenecid/HCTZ-treated group, with sodium excretion increasing from 1.47 ± 0.06 mmole/24 h in vehicle treated animals to 2.35 ± 0.45 mmole/24 h in probenecid/HCTZ group (*p* = 0.03). As shown in **Figure 3A**, the sodium excretion of probenecid treated animals was not significantly different from vehicle treated group (1.47 ± 0.06 mmole/24 h in vehicle vs. 1.46 ± 0.10 in probenecid group). The results of sodium excretion, when adjusted for body weight (mmoles/24 h/g body weight) are as follows: Vehicle 0.007 ± 0.0003, HCTZ 0.006 ± 0.0006, probenecid 0.005 ± 0.004, and probenecid/HCTZ 0.010 ± 0.0002, (*p* = 0.040 vs. probenecid alone or vehicle). The results confirm the robust increase in sodium excretion in rats treated with probenecid/HCTZ in **Figure 3A**.

**Figure 3B** depicts body weight measurements of probenecid-treated vs. probenecid/HCTZ-treated animals. The body weights of vehicle treated group on day 1 of experiments (designated as vehicle/baseline) are used as the initial baseline body weight. The results demonstrate that probenecid-treated animals continued with a normal weight gain throughout the 10 days course of study. However, the probenecid-primed animals that were treated with probenecid/HCTZ combination failed to gain any weight since the start of co-treatment with probenecid and HCTZ. The absence of weight gain in probenecid/HCTZ- compared to probenecid-treated animals (**Figure 3B**) occurs despite their comparable food intakes (0.08 ± 0.004 vs. 0.08 ± 0.003 g food/g body weight/24 h, *n* = 5). Taken together our results suggest that the lack of weight gain in probenecid/HCTZ-treated group reflected enhanced renal fluid loss. Treatment with HCTZ alone for 4 days did not affect the weight gain compared to vehicle-treated rats (both groups showed average weight gain of 4–4.7 g/day).

Enhanced diuresis by probenecid/HCTZ combination was not associated with any decline in kidney function as demonstrated by comparable serum creatinine levels in all experimental groups (**Figure 3C**). Using the standard method of clearance rate of the endogenous marker, creatinine [Ucr (Urine Creatinine) (mg/dl) X Urine Volume/Scr (Serum Creatinine) (mg/dl)], glomerular filtration rates (GFRs) were measured (**Table 1**). Rats treated with Probenecid alone for 10 days at 250 mg/kg/day (0.72 ± 0.07, *n* = 4) or Probenecid/HCTZ combination (0.63 ± 0.08, *n* = 4) showed higher GFR levels when compared to either vehicle (0.45 ± 0.02, *n* = 5) or HCTZ alone (0.53 ± 0.06, *n* = 5) (**Table 1**).

**Table 1:** Measurement of glomerular filtration rate in experimental groups.

	Vehicle	HCTZ	Probenecid (250 mg/kg)	Probenecid + HCTZ
Creatinine Clearance (ml/min)	0.45 ± 0.02	0.53 ± 0.061	0.72 ± 0.07^∗^	0.63 ± 0.08^#^

*^∗^Vehicle (0.45 ± 0.02, *n* = 5) vs. 10 day Probenecid (0.72 ± 0.0723, *n* = 4); *p* = 0.004. ^#^Vehicle (0.45 ± 0.02, *n* = 5) vs. Probenecid + HCTZ (0.63 ± 0.07, *n* = 4); *p* = 0.03.*

### Effect of Probenecid on Pendrin and AQP2 Expression

**FIGURE 4:**
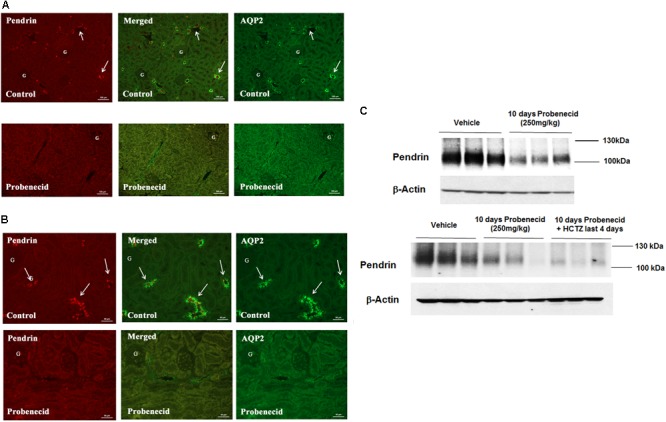
Double immunofluorescence labeling of pendrin and AQP-2 and determination of pendrin abundance in kidneys of animals treated with 250 mg/kg/day of probenecid. **(A)** Pendrin and AQP-2 double immunofluorescent labeling (low magnification). Top: Control. Images are of AQP2 (right) and pendrin (left), with the merged image in the middle panel. Bottom: Probenecid. Effect of 250 mg/kg/day of Probenecid for 10 days on AQP2 (right) and pendrin (left), with merged image in the middle. **(B)** Pendrin and AQP-2 double immunofluorescent labeling (high magnification). Top: Control. Depicted are AQP2 (right) and Pendrin (left), with merged image in the middle. Bottom: Probenecid. Effect of 250 mg/kg of probenecid for 10 days on AQP2 (right) and pendrin (left), with merged image in middle. **(C)** Western blot analysis of pendrin in rats treated with 250 mg/kg probenecid. Top panel: Pendrin abundance in rats treated with 250 mg/kg probenecid. Bottom panel: pendrin abundance in rats treated with probenecid/HCTZ vs. probenecid. The Pendrin abundance, as estimated by scanning densitometry was: Vehicle (0.90 ± 0.06), probenecid (0.46 ± 0.14); *n* = 3, *p* = 0.05 and probenecid/HCTZ (0.34 ± 0.05), *n* = 3; *p* = 0.0004.

To obtain better insight into the mechanism of probenecid/HCTZ-induced diuresis in the 250 mg/kg/day probenecid group, renal expression of pendrin and AQP2 was examined by double immunofluorescence microscopy. As shown in **Figure 4A** (low magnification, top panel), vehicle treated rats showed abundant expression of pendrin and AQP2 in the CCD s. However, the expression of pendrin was reduced in rats treated with 250 mg/kg of probenecid only (**Figure 4A**, low magnification, bottom panel). **Figure 4B** depicts the effect of probenecid alone at a higher magnification and verifies visible downregulation of pendrin by probenecid treatment. **Figure 4C** depicts 2 different sets of experiments examining the expression of pendrin in rats treated with probenecid at 250 mg/kg/day. The western blot in the top panel of **Figure 4C** shows the downregulation of pendrin in probenecid-treated rats. The blot in the Bottom panel of **Figure 4C** confirms the effect of probenecid (250 mg/kg/day) on pendrin expression in a separate experimental group and further demonstrates the robust downregulation of pendrin levels in the probenecid/HCTZ-treated group. These results clearly indicate that probenecid pre-treatment overrides the stimulatory effect of HCTZ on pendrin expression.

### Expression of AQP2, ENaC and NCC in Animals Treated With Probenecid

**FIGURE 5:**
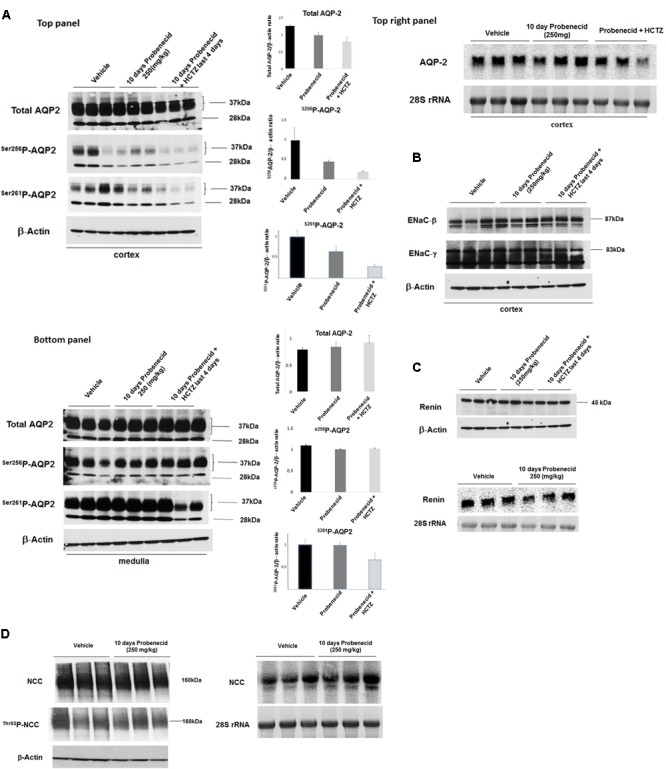
Expression levels of AQP-2, ENaC subunits and renin in kidneys of experimental groups. **(A)** AQP2 expression by western and northern hybridization. The expression levels of AQP2 in the cortex and medulla of Vehicle, probenecid, and probenecid/HCTZ treated animals was assessed by western blot analysis. There were no significant differences in Total AQP2, ^Ser256^p-AQP2 or ^Ser261^p-AQP2 levels in the medulla of Vehicle versus probenecid (*p* = 0.41; *p* = 0.40; or *p* = 0.94 respectively) or probenecid/HCTZ (*p* = 0.12; *p* = 0.98; or *p* = 15 respectively) animals when the densitometry values were normalized against β-Actin. There were no significant differences between Total AQP2, ^Ser256^p-AQP2 or ^Ser261^p-AQP2 in the cortices of Vehicle versus probenecid (*p* = 0.07, *p* = 0.20, and 0.15; respectively). However, there was a significant difference between the cortices of Vehicle versus probenecid + HCTZ in Total AQP2 (*p* = 0.0001, *n* = 3) and ^Ser261^p-AQP2 (*p* = 0.01, *n* = 3) (**A**, left top and bottom panels). Northern blot analyses (**B**, right top panel) were performed on RNA samples from the cortices of probenecid treated (250 mg/kg) animals. As indicated, mRNA expression levels were not significantly different in vehicle and probenecid treated rats (*p* = 0.33). **(B)** ENaC Western blot. The expression levels of ENaC subunits, alpha and gamma, in the cortex were examined using subunit-specific antibodies. As indicated, both subunits showed comparable expression levels in experimental groups. **(C)** Renin Western and Northern blots. Protein and RNA values were quantified and normalized based on β-Actin or 28S rRNA, respectively. Protein abundance (top panel) and RNA expression (bottom panel) of renin in kidney cortex of Vehicle, probenecid-treated (250 mg/kg/day) and probenecid/HCTZ-treated groups were comparable (*p* = 0.293, *n* = 3/group for Western; *p* = 0.34, *n* = 3/group for Northern) **(C)**. Student’s unpaired *t*-test was used for statistical analysis. **(D)** NCC Western and Northern Blots. The NCC mRNA expression levels (**D**, right) were comparable in both Vehicle (0.94 ± 0.09, *n* = 3) and in 250 mg/kg probenecid treatment animals (0.96 ± 0.1, *n* = 3; *p* = 0.90). Western blot analysis of total NCC and phosphorylated NCC were comparable in Control and 250 mg/kg/day probenecid treated groups. Left upper panel (NCC): Vehicle (1.02 ± 0.09, *n* = 3) vs. probenecid (1.02 ± 0.02, *n* = 3); *p* = 0.98; and left middle panel (^Thr53^p-NCC): Vehicle (0.87 ± 0.13, *n* = 3) vs. probenecid (0.87 ± 0.03, *n* = 3); *p* = 0.97.

The immunofluorescence studies in the previous section (**Figure 4**) suggested a reduction in AQP2 expression levels by probenecid pre-treatment at 250 mg/kg/day. Further, there was a marginal increase in urine output and reduction in urine osmolality in the aforementioned probenecid-treated alone group (**Figures 2A,C**). These findings may point to a defect in water salvage mechanisms in the collecting duct (reflecting compromised AQP2 expression and/or function). In the next series of experiments, we examined the expression of AQP2 in the cortex and medulla of control, probenecid and probenecid/HCTZ-treated animals. Accordingly, the abundance of total AQP2, AVP-stimulated **^Ser256^**p-AQP2 and AVP-independent **^Ser261^**p-AQP2 was examined. Western blot analysis of kidney cortex extracts revealed that the expression levels of total AQP2, ^Ser256^p-AQP2, and ^Ser261^p-AQP2 were visibly reduced in kidneys of probenecid- and probenecid/HCTZ co-treated animals compared to their vehicle treated counterparts (**Figure 5A**, top panel). The expression of total AQP-2, ^Ser256^p-AQP2, and ^Ser261^p-AQP2 in the medulla of probenecid and probenecid/HCTZ-treated rats remained unchanged (**Figure 5A**, bottom panel). Northern blot analyses (**Figure 5A**, top right panel) performed on cortex samples from probenecid treated (250 mg/kg/day) rats showed no significant difference in AQP2 expression when normalized for 28S rRNA levels (Vehicle 1.05 ± 0.08 vs. probenecid 250 mg/kg/day 1.21 ± 0.13, *n* = 3; *p* = 0.33).

Expression levels of ENaC subunits, beta and gamma, in the cortex were comparable in Vehicle, probenecid and probenecid/HCTZ groups (**Figure 5B**). Protein (**Figure 5C**, top) and mRNA (**Figure 5C**, bottom) expression levels of renin in kidney cortex of Vehicle, probenecid-treated (250 mg/kg/day) and probenecid/HCTZ-treated groups were comparable (*p* = 0.29, *n* = 3/group and *p* = 0.34, *n* = 3/group for western and northern blot analyses, respectively) (**Figure 5C**, top and bottom panels). The expression levels of NCC mRNA (**Figure 5D**, right panel) were comparable in both Vehicle and in 250 mg/kg/day probenecid treated rats (*n* = 3; *p* = 0.90). Western blot analysis of total NCC and phosphorylated NCC showed comparable levels in control and probenecid treated groups (*n* = 3; *p* = 0.10 for NCC and p-NCC, respectively) (**Figure 5D**, left panel).

### Systemic Acid Base and Electrolyte Homeostasis in Probenecid- and Probenecid/HCTZ-Treated Rats

The effect of probenecid, HCTZ or probenecid/HCTZ-cotreatment on systemic acid base homeostasis and electrolytes was determined. The results depicted in **Table 2** show comparable arterial pH, HCO_3_^-^, as well as serum Na^+^ levels. There was a significant difference between pCO_2_ levels (in mm Hg) in Vehicle versus probenecid and probenecid/HCTZ treated animals (Vehicle 61.84 ± 1.78, *n* = 5 vs. probenecid 53.34 ± 1.12, *n* = 5; *p* = 0.004) (Vehicle 61.84 ± 1.78, *n* = 5 vs. probenecid/HCTZ 55.44 ± 1.68, *n* = 5; *p* = 0.03). In addition, there was also a significant difference in serum K^+^ levels between Vehicle versus rats treated with probenecid/HCTZ (Vehicle 4.98 ± 0.24, *n* = 5 vs. probenecid/HCTZ 4.24 ± 0.15, *n* = 5; *p* = 0.03), as well as probenecid versus probenecid/HCTZ (probenecid 5.18 ± 0.10, *n* = 5 vs. probenecid/HCTZ 4.24 ± 0.15, *n* = 5; *p* = 0.0007). Finally, there was a significant difference between serum Ca^2+^ levels in Vehicle versus probenecid treated animals (Vehicle 1.27 ± 0.03, *n* = 5 vs. probenecid 1.16 ± 0.01, *n* = 5; *p* = 0.001).

**Table 2:** Systemic acid base and electrolyte homeostasis.

	Arterial pH	pCO_2_ (mmHg)	HCO_3_^-^ (mM/L)	Serum Na^+^ (mEq/L)	Serum K^+^ (mEq/L)	Serum Ca^2+^ (mM/L)]
Vehicle	7.32 ± 0.01	61.84 ± 1.78	31.86 ± 0.39	135.20 ± 0.73	4.98 ± 0.24	1.29 ± 0.03
Probenecid (250 mg/kg)	7.36 ± 0.02	53.34 ± 1.12^∗^	30.32 ± 0.58	136.60 ± 0.24	5.18 ± 0.10	1.16 ± 0.01^%^
HCTZ	7.34 ± 0.02	59.12 ± 1.70	32.22 ± 1.28	136.20 ± 0.73	4.74 ± 0.16	1.24 ± 0.02
Probenecid + HCTZ	7.36 ± 0.02	55.44 ± 1.68^#^	31.66 ± 1.10	136.60 ± 0.81	4.24 ± 0.15^+,∧^	1.19 ± 0.05

*^∗^Vehicle pCO_*2*_ (61.84 ± 1.78, *n* = 5) vs. probenecid pCO_*2*_ (53.34 ± 1.12, *n* = 5); *p* = 0.003. ^#^Vehicle pCO_*2*_ (61.84 ± 1.78, *n* = 5) vs. probenecid + HCTZ pCO_*2*_ (55.44 ± 1.68, *n* = 5); *p* = 0.03. ^+^Vehicle K^+^ (4.98 ± 0.24, *n* = 5) vs. probenecid + HCTZ K^+^ (4.24 ± 0.15, *n* = 5); *p* = 0.03. ^∧^Probenecid K^+^ (5.18 ± 0.10, *n* = 5) and probenecid + HCTZ (4.24 ± 0.15, *n* = 5); *p* = 0.0007. ^%^Vehicle Ca^*2*+^ (1.27 ± 0.03, *n* = 5) vs. probenecid (1.16 ± 0.01, *n* = 5); *p* = 0.001.*

**FIGURE 6:**
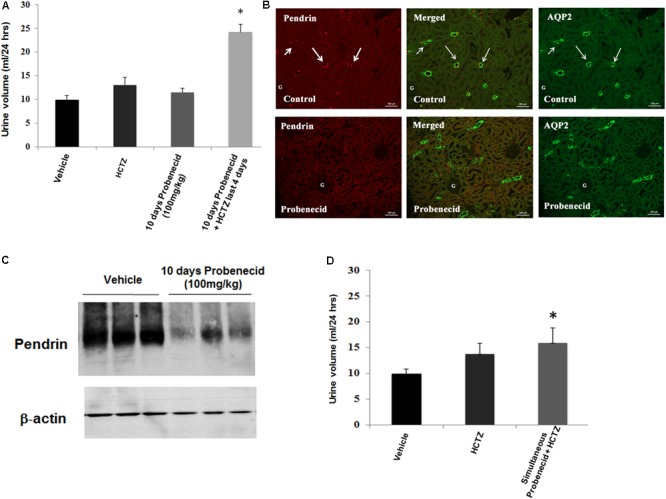
Effect of probenecid at 100 mg/kg, with or without HCTZ, on urine volume and pendrin expression. **(A)** Effect of probenecid pre-treatment on HCTZ-induced diuresis. Data depicts urine output in rats pre-treated with Probenecid at 100 mg/kg for 6 days followed by co-treatment with 100 mg/kg Probenecid and HCTZ for an additional 4 days. When adjusted for body weight, the results, expressed as urine volume/24 h/body weight were as follows: Vehicle (0.05 ± 0.004, *n* = 12) vs. probencid (100 mg) ± HCTZ (0.11 ± 0.008, *n* = 7); *p* = 0.000001. ^∗^ denotes significance between Vehicle vs. Probenecid/HCTZ co-treatment. **(B)** Double immunofluorescence labeling with pendrin and AQP-2. Top panel: Control. AQP2 (right) and Pendrin (left), with merged image in the middle panel. Bottom: probenecid. Effect of 100 mg/kg of probenecid for 10 days on AQP2 (right) and pendrin (left), with merged images in middle. **(C)** Western blot analysis of pendrin in probenecid pre-treated rats at 100 mg/kg/day. Western blots indicate significant reduction in the expression of pendrin in rats pre-treated with probenecid at 100 mg/kg/day for 6 days. Vehicle (1.01 ± 0.02, *n* = 3) vs. 10 days probenecid (100 mg/kg) (0.56 ± 0.11, *n* = 3; *p* = 0.02). **(D)** Effect of simultaneous treatment of probenecid and HCTZ without probenecid priming on urine volume. The magnitude of daily urine volume in simultaneous co-treatment of 100 mg/kg/day of probenecid and HCTZ without probenecid priming was not significantly different when compared to HCTZ-treated animals but was significant vs. vehicle treated rats. When adjusted for body weight, the results, expressed as urine volume/24 h/body weight were as follows: HCTZ (0.06 ± 0.01, *n* = 5) vs. Simultaneous probenecid + HCTZ (0.07 ± 0.01, *n* = 4; *p* = 0.42). ^∗^ denotes significance between Vehicle and Simultaneous treatment of Probenecid/HCTZ. However, there was no significant difference between simultaneous treatment of Probenecid/HCTZ and HCTZ alone.

In the last series of experiments, we examined the effect of probenecid at 100 mg/kg/day, with or without HCTZ, on salt and water excretion. **Figure 6A** depicts the effect of probenecid at 100 mg/kg and HCTZ, alone or in combination, on urine output. Daily treatment with probenecid (100 mg/kg) for up to 10 days had no significant effect on diuresis in rats, with baseline urine output of 9.93 ± 0.93 ml/day increasing to 11.45 ± 0.99 ml/day after 10 days (*p* = 0.5, *n* = 7). Daily injection with HCTZ alone for 4 days increased the urine output to 13.06 ± 1.53 ml/day (data shown for day 4 of HCTZ treatment). However, in rats that were primed with 100 mg/kg probenecid for 6 days, daily treatment with HCTZ and probenecid for 4 additional days (total duration of 10 days for probenecid and 4 days for HCTZ) increased the urine output to 24.23 ± 1.71 ml/day on day 4 of HCTZ/probenecid co-administration. This increase was significant compared to probenecid (*p* = 0.03, *n* = 4) or HCTZ (*p* = 0.007, *n* = 7) treatment alone.

### Effect of Probenecid at 100 mg/kg on Pendrin Expression

The expression of pendrin in rats treated with 100 mg/kg/day of probenecid for 10 days was examined by double immunofluorescence labeling using anti-pendrin and AQP2 antibodies. As indicated, pendrin expression was visibly reduced in kidneys of rats treated with 100 mg/kg of probenecid (**Figure 6B**, bottom panel vs. top panel).

In order to further examine the effect of probenecid on pendrin expression, western blot analysis was performed on membrane proteins isolated from cortices of probenecid treated rats. **Figure 6C** shows significant reduction in pendrin abundance (1.01 ± 0.02, *n* = 3 vs. 0.56 ± 0.11, *n* = 3; *p* = 0.015) in rats treated with 100 mg/kg/day of probenecid compared to vehicle treated controls.

### The Maximal Diuretic Effect of Probenecid/HCTZ Requires Pre-treatment With Probenecid

The role of probenecid pre-treatment on the magnitude of diuresis in the probenecid/HCTZ co-treatment group was examined by simultaneous treatment of rats with probenecid (100 mg/kg) and HCTZ (4 mg/100 g BW) for 2 days with no probenecid pre-treatment. The results are included in **Figure 6D** and indicates the urine output of 15.87 ± 2.91 ml/day in rats treated simultaneously with probenecid plus HCTZ without probenecid pre-treatment was not significantly higher than the urine volumes in animals treated with HCTZ alone (*p* = 0.283, *n* = 4). These results indicate that pre-treatment with probenecid is a pre-requisite for enhancement of HCTZ-induced diuresis.

### Effect of Probenecid on HCTZ Excretion

Renal excretion of HCTZ in the presence or the absence of probenecid injection was determined by an ELISA. Our results indicate that in rats treated with probenecid, HCTZ excretion is reduced compared to those treated with HCTZ alone (**Table 3**), confirming published literature on the interaction of probenecid with the OATs that mediate thiazide secretion into the proximal tubule.

**Table 3:** Effect of probenecid on HCTZ excretion.

Treatment	HCTZ excretion (ng/24h)
HCTZ alone	12962.00 ± 2944.89
Probenecid + HCTZ	7074.43 ± 503.54

## Discussion

The proper selection of diuretics for the treatment of fluid overloaded states is a challenging decision and is determined by the etiology and severity of fluid overload, as well as the strength, mechanism of action and possible side effects of diuretic agent(s). Loop diuretics such as bumetanide or furosemide, which inhibit the apical Na–K–2Cl co-transporter (NKCC2) in the thick ascending limb, are powerful natriuretic agents but have numerous side effects, including severe hypokalemia, volume depletion and metabolic alkalosis (Schultheis et al., 1998; Gamba, 2005; Sica et al., 2011). Amiloride analogs, which are inhibitors of the sodium channel ENaC in the collecting duct, are associated with hyperkalemia, hence they may be used in conjunction with the loop diuretics or thiazides (Emanuelsson and Paalzow, 1988; Hesselink et al., 1999). Thiazides are the most widely used diuretic for mild hypertension and as a combination therapy for moderate hypertension (Ellison, 2003; Gamba, 2005; Alshahrani et al., 2017). They have also been used in conjunction with loop diuretics or ENaC inhibitors for the treatment of severe fluid overload. The use of thiazides and loop diuretics are associated with hyperuricemia, which can adversely affect kidney and cardiac functions.

Thiazide derivatives are specific inhibitors of NCC, which is the main salt absorbing transporter in the DCT (Schultheis et al., 1998; Ellison, 2003; Loffing et al., 2004; Gamba, 2005). Despite being a strong inhibitor of NCC, thiazide derivatives in general are mild diuretics, likely due to compensatory salt absorption by transporters in other nephron segments. Both the sodium channel ENaC and Cl^-^/HCO_3_^-^ exchanger pendrin are activated in the CNT and CCD; therefore, blunting the diuresis caused by the inhibition or inactivation of NCC (Patel-Chamberlin et al., 2016). Inactivation or inhibition of pendrin provokes robust diuresis by HCTZ, a potent thiazide analog (Soleimani et al., 2012; Zahedi et al., 2013; Alshahrani et al., 2017).

The most salient feature of the current studies was the profound diuresis by HCTZ and probenecid, which required pre-treatment with probenecid for its maximal effect. Probenecid is not a known diuretic by itself and has not previously been shown to increase urine output. It has multiple effects on various systems and nephron segments, ranging from the inhibition of OATs in the proximal tubule (leading to uric acid excretion) to the inhibition of the ATP transporter Pannexin 1 in the proximal tubule and collecting duct (Halpin and Renfro, 1996; Silverman et al., 2008; Bach and Simkin, 2014; Richette et al., 2014). Our studies demonstrate that treatment with probenecid at either 250 or 100 mg/kg body weight for 10 days causes significant perturbation in the expression of transporters in the collecting duct, including the downregulation of pendrin and, at the higher probenecid dosing (250 mg/kg/day), the AQP2. The downregulation of pendrin enhances salt and water excretion by thiazides, consistent with the indispensable role of pendrin in compensatory salt absorption in response to inactivation/inhibition of NCC (Soleimani et al., 2012; Zahedi et al., 2013; Patel-Chamberlin et al., 2016; Alshahrani et al., 2017). We did not examine the effect of probenecid on the expression of the sodium dependent Cl^-^/HCO_3_^-^ exchanger, NDCBE (SLC4A8) (Leviel et al., 2010), as a recent report indicates its absence in CCD (Chen et al., 2017).

The mechanism of pendrin downregulation by probenecid remains speculative. While the expression of pendrin protein was significantly reduced, its mRNA expression levels were not affected by probenecid, consistent with a post transcription effect, such as impairing the trafficking of the transporter to the plasma membrane. At 100 mg/kg/day, probenecid had no significant effect on AQP2 expression; however, at 250 mg/kg/day it significantly reduced the expression of AQP2 in the CCD (**Figure 5A**). The expression of AQP2 in the medullary collecting ducts remained unchanged in response to probenecid treatment (**Figure 5A**). The downregulation of AQP2 by probenecid was not observed at the mRNA levels, consistent with a post-transcriptional effect in CCD. The effect of probenecid at 250 mg/kg/day on the CCD transporters AQP-2 and Pendrin is specific and did not involve ENaC expression. Whether the effect of probenecid on AQP2 is mediated via its ability to inhibit Pannexin 1, which is an ATP conduit expressed on the apical membrane of the collecting duct (and proximal tubule) and plays a role in purinergic signaling (Silverman et al., 2008), remains to be determined. Further, whether the inhibition of Pannexin1 by probenecid and the corresponding impairment in ATP release could modulate ENaC activity and contribute to enhanced diuresis in our experimental models remains to be determined.

Recent studies indicate that probenecid possesses a positive ionotropic effect, specifically after reperfusion injury, and can increase cardiac output (Koch et al., 2012; Rubinstein et al., 2014). In detailed studies, probenecid was found to enhance the cardiac contractility via activation of TRPV2 channels in the cardiomyocytes secondary to SR release of Ca^2+^ (Koch et al., 2012; Rubinstein et al., 2014). It is plausible that enhanced cardiac contractility by probenecid increases kidney GFR, resulting in increased delivery of solutes to the distal nephron. Examination of kidney function in the present study indicates that GFR is enhanced in probenecid-treated groups (either Probenecid alone or Probenecid plus HCTZ) but not in animals treated with HCTZ alone. It is plausible that increased delivery of solutes to the distal nephron coupled to the downregulation of pendrin and AQP2 enhances the diuresis caused by HCTZ. The tubular secretion of creatinine into proximal tubule lumen may be reduced in the presence of probenecid or in animals with the inactivation of OATs (Darling and Morris, 1991; Vallon et al., 2012) which could increase serum creatinine and erroneously affect the estimated GFR. It is noteworthy to mention that any interference of probenecid with creatinine secretion into the lumen of proximal tubule, as indicated in rats (Darling and Morris, 1991) or in animals with OAT inactivation (Vallon et al., 2012) should actually result in underestimation rather than overestimation of GFR.

As a uricosuric agent, probenecid predominantly affects the transport of organic anions, such as urate, in the proximal tubule (Bach and Simkin, 2014; Richette et al., 2014). By blocking the reuptake of urate, probenecid increases uric acid excretion. Diuretics, such as HCTZ, are known to cause hyperuricemia by competing with urate for transport and secretion via OATs in the proximal tubule. It is therefore, highly plausible that the uricosuric action of probenecid will diminish the HCTZ-induced hyperuricemia, which is a common side effect in patients receiving this medication.

There are no reports examining the effect of probenecid on salt transporters or excretion. In a study on hypercalciuric patients, probenecid did not show any significant effect on salt excretion by acute treatment with thiazides (Garcia and Yendt, 1970). There are also no studies examining the chronic interaction of thiazides and Probenecid on salt excretion in normal volunteers or in animal models. Thiazide derivatives are excreted unchanged in the urine through glomerular filtration and secretion into the proximal tubule (Garcia and Yendt, 1970). Probenecid inhibits the tubular secretion of thiazides by interfering with their transport via OATs, consequently reducing delivery of thiazides to the lumen of DCT, the active site for the inhibition of NCC by thiazides. Our results (**Table 3**) also confirms reduced excretion of HCTZ in probenecid pre-treated rats. A reduction in the diuretic effect of thiazides is expected if their delivery to the lumen of DCT is diminished by probenecid. On the other hand, by interfering with their tubular secretion, probenecid can prolong the half-life of thiazides thereby enhancing their efficacy. However, to our knowledge no previous study has either examined or indicated any alteration in diuretic efficacy of thiazides by probenecid.

The downregulation of pendrin in the absence of HCTZ (**Figures 4, 6**) clearly shows that the priming effect of the kidney by probenecid is unrelated to its interaction with HCTZ. Furthermore, our observation that the magnitude of diuresis in HCTZ treated animals (13.7 ± 2.13 ml/24 h) was not significantly different from those that received simultaneous treatment with HCTZ and probenecid without priming with the latter (15.86 ± 2.91 ml/24 h) (**Figure 6D**), strongly argues that the stimulatory diuretic effect of probenecid on HCTZ-induced diuresis requires pre-treatment with probenecid and is independent of any interaction between the two medications/drugs.

In conclusion, probenecid down-regulates pendrin, leaving the thiazide sensitive transporter NCC, as the major salt absorbing transporter in the distal nephron. Probenecid, at a higher dose, also down-regulates AQP2 in the CCD. We propose that priming with probenecid followed by co-treatment with probenecid/thiazide derivatives enhances salt and water excretion; and may therefore be a powerful regimen for the treatment of fluid overloaded states such as congestive heart failure.

## Materials and Methods

### Animal Models and Balance Studies

Male Sprague Dawley rats (7–8 weeks of age) were placed in metabolic cages and divided into three treatment groups (**Figure 1**). These consisted of the following groups (see **Table 4**).

**Table 4:** Explanation of treatments, dosages, route of administration, and durations for experimental groups.

Group	Treatment/route of administration	Dosage	Volume injected	Duration
Control (*n* = 5)	Vehicle	Vehicle given at the same volume and same route	1 μl/g BW	10 days
HCTZ (*n* = 5)	HCTZ (s.c.)	40 mg/kg BW	1 μl/g BW	4 days
Probenecid (*n* = 5)	Probenecid (i.p.)	250 mg/kg or 100 mg/kg BW	1 μl/g BW	10 days
Probenecid + HCTZ (*n* = 5)	Probenecid (i.p.) plus hydrochlorothiazide (s.c.)	250 mg/kg or 100 mg/kg probenecid + 40 mg/kg HCTZ	1 μl/g BW	Probenecid priming for 6 days, then co-treatment with HCTZ for 4 days.

Vehicle was administered to control group for the same number of days as treatments in experimental groups. Hydrochlorothiazide (HCTZ) was given subcutaneously (s.c.) at a dose of 40 mg/kg and probenecid was injected intraperitoneally (i.p.) at a dose of either 100 mg/kg or 250 mg/kg. The vehicles were administered at the same volume/dosage as the treatment. HCTZ (200 mg) was dissolved in 1 ml of 100% ETOH + 4 ml propylene glycol to generate a 40 mg/ml solution. Probenecid was dissolved in 5 ml of 0.5M NaOH + 15 ml of PBS to generate an either 250 mg/ml or 100 mg/ml solution. The volume injected for each treatment was 1 μl/g BW. Each experiment was repeated twice. Rats were placed in metabolic cages (Techniplast #3700M071, West Chester, PA, United States) for balance studies where they were acclimated for 24 h before samples were collected. Balance studies (24 h water intake, urine output, food intake, body weight, and urine osmolality) were measured daily. Twenty four hour water and food intake were determined by weighing samples each day and subtracting this from the previous day’s allotment. Urine was collected daily under mineral oil to avoid evaporation. At the end of the experiments, animals were euthanized and their kidneys were harvested. Blood was collected via direct heart puncture in heparinized needles and was later centrifuged to collect serum for analysis.

Animals were housed and cared for in accordance with the Institutional Animal Care and Use Committee (IACUC) at the University of Cincinnati. All animal handlers were IACUC-trained. Animals had access to food and water *ad libitum*, were housed in humidity, temperature, and light/dark controlled rooms, and were inspected daily. Animals were euthanized with the use of excess anesthetics (pentobarbital sodium) according to institutional guidelines and approved protocols. All animal studies described in this manuscript were approved by the University of Cincinnati IACUC Review Board.

### Immunofluorescence Labeling

Animals were euthanized with an overdose of pentobarbital sodium, and kidneys were removed, cut in tissue blocks, and fixed in a 4% formaldehyde solution overnight at 4°C. The tissue was then transferred to 70% ethanol and embedded in paraffin, and 5-μm sections were cut and stored until used. Double-immunofluorescence labeling using anti-pendrin and anti-AQP2 antibodies were performed as previously described (Soleimani et al., 2011; Xu et al., 2011). After 24 h incubation at 4 degrees in primary antibodies, sections were labeled with either AlexaFluor goat-anti-rabbit IgG or goat-anti-mouse IgG (Life Technologies, Eugene, OR, United States). Fluorescent images were obtained on a Zeiss Axio Imager.M2 with Zen Software (Thornwood, NY, United States).

### Western Blotting

For Western blot analysis, tissue was homogenized in a 250 mM sucrose solution containing 10 mM Triethanolamine and spun in an Ultracentrifuge (Beckman, Brea, CA, United States) to isolate membrane-bound proteins to isolate membrane-bound proteins. Proteins were then size-fractionated by SDS/PAGE (90 μg/lane), transferred to nitrocellulose membrane. The blots were examined for the expression of renin, Pendrin, AQP2, ^Ser256^p-AQP2, ^Ser261^p-AQP2, and Actin. The secondary antibodies were anti-sheep IgG conjugated to horseradish peroxidase for renin, anti-goat IgG conjugated to horseradish peroxidase for AQP2 and actin and anti-rabbit IgG conjugated to horseradish peroxidase for ^Ser256^p-AQP2 and ^Ser261^p-AQP2. The bands were visualized using the HRP Chemiluminescence Kit (Invitrogen, Carlsbad, CA, United States) and images were captured on light-sensitive imaging film (MidSci, St. Louis, MO, United States).

### Antibodies

Pendrin antibodies for immunofluorescence labeling were generated in our laboratory as described (Amlal et al., 2010). The Pendrin antibodies for Western blotting were generous gifts from Dr. Peter Aronson (Knauf et al., 2001). The monoclonal antibody against AQP2 was kindly provided by Dr. Ann Blanchard (Dr. Anne Blanchard, Centre d’Investigations Cliniques, Hôpital Européen Georges Pompidou, Paris, France). Antibodies against renin (MyBiosource, #MBS315812, San Diego, CA, United States), Actin and polyclonal AQP2 (Santa Cruz Biotechnology, Santa Cruz, CA, United States), ^Ser256^p-AQP2 (Assay Biotech, Sunnyvale, CA, United States), and ^Ser261^p-AQP2 (Abnova, Taipei, Taiwan). ENaC-β, ENaC-γ, and NCC antibodies were purchased from StressMarq Biosciences (British Columbis, Canada).

### Northern Blot Analysis

Northern hybridizations were performed according to established protocols (Soleimani et al., 2001; Amlal et al., 2010). Briefly, total cellular RNA was extracted from kidneys of rats, quantitated spectrophotometrically, and stored at -80°C. Total RNA samples (30 μg/lane) were fractionated on a 1.2% (g/dl) agarose-formaldehyde gel, transferred to Magna NT nylon membranes, cross-linked by UV light, and baked. Hybridization was performed according to established methods. The membranes were washed, blotted dry and exposed to a PhosphorImager screeen. The signal strength of hybridization bands was quantitated by densitometry using ImageQuaNT software.

Gene specific, PCR amplified rat cDNA fragments were used as specific probes for renin, AQP-2 and NCC. For NCC, a DNA fragment spanning the nucleotides 481–959 of mouse NCC cDNA (Genebank # NM_001205311) was generated using the following primers: *GTGATCCTCTACCTGCGACTC* (sense) and *TTGTCTTCAGATGCTGGGATC* (antisense). For renin, a PCR fragment encoding nucleotides 291–600 (accession number NM_031192) and for AQP2, a fragment encoding nucleotides 102–397 (Accession number: BC128705.1) were used.

### Blood Acid Base, Chemicals and Electrolyte Analysis

Serum concentration of Na^+^, K^+^, Ca^2+^, and HCO_3_^-^ were quantified using an i-STAT-1 analyzer with i-STAT EG7+ cartridges (Abbott Laboratories, Abbott Park, IL, United States). Serum creatinine levels were quantified using QuantiChrom Creatinine Assay Kit (BioAssay Systems, Hayward, CA, United States). Urine was collected under mineral oil.

### Urine Osmolality and Electrolyte Excretion

Urine osmolality values were obtained via a micro-osmometer (Advanced Instruments, Norwood, MA, United States). Urine sodium, chloride and potassium concentrations were measured with “EasyLyte Plus; Na/K/Cl” instrument from Medica Corp (Bedford, MA, United States). Urine pH was measured with a pH meter (Accumet Basic AB15, Fisher Scientific, Hanover Park, IL, United States).

### HCTZ Excretion in the Urine

Twenty four hour urine HCTZ excretion rates were measured using the HCTZ ELISA Kit from Neogen (Lexington, KY, United States).

### Statistics Analysis

The results for blood and urine chemistry data are presented as means ± SE. Statistical significance between 2 or more experimental groups was determined by Student’s *t-*test or ANOVA, respectively, and *P* < 0.05 was considered significant. Unless indicated, 5–7 animals from each group were used for metabolic cage, urine and blood chemistries and expression (northern, western or immunofluorescence) studies.

## Author Contributions

SB: performed the experiments, acquired and prepared immunofluorescence labeling images, and assisted with manuscript preparation. JX: performed the experiments including northern and western blots. KZ: performed the experiments, including injections of animals. MB: collected urine samples in animals in metabolic cages and analyzed salt excretion. MS: conceptualized the experiments, analyzed the data, and wrote the manuscript.

## Conflict of Interest Statement

The authors declare that the research was conducted in the absence of any commercial or financial relationships that could be construed as a potential conflict of interest.
